# 2,4-Dinitro-1-naphthol

**DOI:** 10.1107/S1600536810045034

**Published:** 2010-11-06

**Authors:** Abdul Rauf Raza, Aeysha Sultan, M. Nawaz Tahir, Amra Rubab

**Affiliations:** a Department of Chemistry, University of Sargodha, Sargodha, Pakistan; b Department of Physics, University of Sargodha, Sargodha, Pakistan

## Abstract

In the title compound, C_10_H_6_N_2_O_5_, the two fused rings are almost co-planar, with an r.m.s. deviation of 0.0163 Å. The nitro groups are oriented at dihedral angles of 2.62 (11) and 44.69 (11)° with respect to the plane of the parent fused rings. Intra­molecular O—H⋯O and C—H⋯O hydrogen bonds complete *S*(6) ring motifs. In the crystal, mol­ecules are linked into chains along [101] by inter­molecular O—H⋯O hydrogen bonds. π–π inter­actions [centroid–centroid distances = 3.6296 (15), 3.8104 (15) and 3.6513 (14) Å] might play a role in stabilizing the structure.

## Related literature

For background to estrogens, see: Schwartz *et al.* (1995 ▶); O’Donnell *et al.* (2001) ▶. For related structures, see: Filipenko *et al.* (2001 ▶); Rozycka-Sokolowska *et al.* (2004 ▶). For graph-set notation, see: Bernstein *et al.* (1995 ▶). For π–π inter­actions, see: Janiak (2000 ▶).
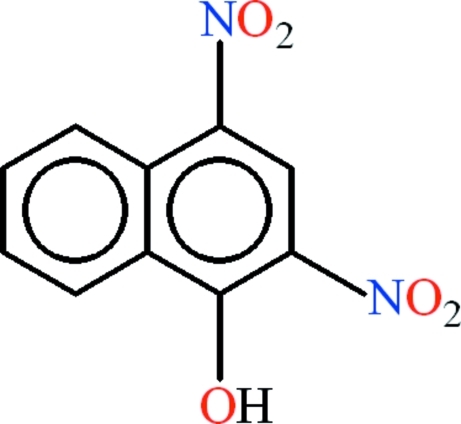


## Experimental

### 

#### Crystal data

C_10_H_6_N_2_O_5_
*M* *_r_* = 234.17
Monoclinic, 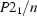

*a* = 7.0512 (10) Å
*b* = 16.3541 (19) Å
*c* = 8.7988 (10) Å
β = 111.452 (6)°
*V* = 944.4 (2) Å^3^
*Z* = 4
Mo *K*α radiation
μ = 0.14 mm^−1^
*T* = 296 K
0.32 × 0.14 × 0.12 mm

#### Data collection

Bruker Kappa APEXII CCD diffractometer
Absorption correction: multi-scan (*SADABS*; Bruker, 2005 ▶) *T*_min_ = 0.978, *T*_max_ = 0.982
6868 measured reflections
1684 independent reflections
1058 reflections with *I* > 2σ(*I*)
*R*_int_ = 0.052

#### Refinement

*R*[*F* ^2^ > 2σ(*F* ^2^)] = 0.042
*wR*(*F* ^2^) = 0.119
*S* = 1.00
1684 reflections
155 parameters
H-atom parameters constrained
Δρ_max_ = 0.18 e Å^−3^
Δρ_min_ = −0.17 e Å^−3^

### 

Data collection: *APEX2* (Bruker, 2009 ▶); cell refinement: *SAINT* (Bruker, 2009 ▶); data reduction: *SAINT*; program(s) used to solve structure: *SHELXS97* (Sheldrick, 2008 ▶); program(s) used to refine structure: *SHELXL97* (Sheldrick, 2008 ▶); molecular graphics: *ORTEP-3 for Windows* (Farrugia, 1997 ▶) and *PLATON* (Spek, 2009 ▶); software used to prepare material for publication: *WinGX* (Farrugia, 1999 ▶) and *PLATON*.

## Supplementary Material

Crystal structure: contains datablocks global, I. DOI: 10.1107/S1600536810045034/dn2615sup1.cif

Structure factors: contains datablocks I. DOI: 10.1107/S1600536810045034/dn2615Isup2.hkl

Additional supplementary materials:  crystallographic information; 3D view; checkCIF report

## Figures and Tables

**Table 1:** Hydrogen-bond geometry (Å, °)

*D*—H⋯*A*	*D*—H	H⋯*A*	*D*⋯*A*	*D*—H⋯*A*
O5—H5*A*⋯O1^i^	0.82	2.53	3.006 (3)	118
O5—H5*A*⋯O4	0.82	1.87	2.573 (2)	142
O5—H5*A*⋯N2	0.82	2.47	2.892 (3)	113
C5—H5⋯O1	0.93	2.35	2.902 (3)	118

Symmetry code: (i) 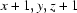.

**Fig. 1.**
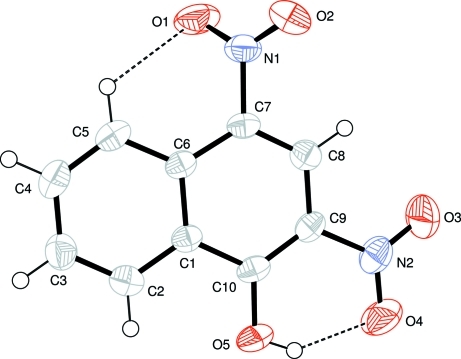
View of the title compound with the atom numbering scheme. The thermal ellipsoids are drawn at the 50% probability level. H-atoms are shown by small circles of arbitrary radii. The dotted line indicate the intramolecular H-bond.

**Fig. 2.**
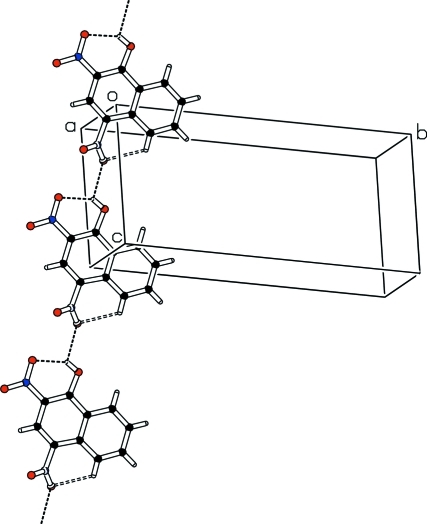
The partial packing (PLATON; Spek, 2009) which shows that molecules form polymeric chains.

## supplementary crystallographic information

### Comment

Estrogens have been found to have maintenance effects on bone, brain, skin, cardiovascular system (Schwartz *et al.*, 1995). In spite of their beneficial biological effects, estrogens are suspected to have relationship with the risk of cancer and thromboemetic diseases (O'Donnell *et al.*, 2001). The title compound was obtained as an interesting side product while synthesizing substituted 1-tetralone as AB ring of the estrogen skeleton which will bear substituent at positions not found in nature.

The title compound (I) is related to the published crystal structures of 1-naphthalenol (Rozycka-Sokolowska *et al.*, 2004) and hydroxonium 2,4-dinitro-1-hydroxy-7-sulfonatonaphthalene monohydrate (Filipenko *et al.*, 2001).

In (I), the two fused rings (C1—C10) are neraly planar with the largest deviation being 0.033 (2)Å at C10 (Fig. 1). The hydroxyl O5 atom is only deviating by 0.129 (2)Å from the mean plane. Owing to the intramolecular O5–H···O4 hydrogen bond (Table 1), the O3 and O4 atoms are only slightly displaced from the mean plane of the fused rings by -0.074 (2)Å and 0.023 (2) Å, respectively. The other nitro group is twisted by 44.72 (12)° with respect to the fused rings.

Strong intramolecular H-bondings of O—H···O and C—H···O types (Table 1, Fig. 1) complete S(6) ring motifs (Bernstein *et al.*, 1995). The molecules are stabilized in the form of polymeric chains due to intermolecular O—H···O hydrogen bonds (Table 1, Fig. 2). Slippest weak π–π interactions (Table 2) might play a role in stabilizing the packing.

### Experimental

The 1-tetralone (1 ml, 1.1 g, 1 eq) was added to a chilled and well stirred nitrating mixture containing H_2_SO_4_ (2 ml, 0.57 g, 2 eq) and HNO_3_ (0.5 ml, 0.7 g, 1.5 eq). The reaction mixture was then neutralized and extracted with EtOAc (3 × 25 ml) and the combined organic extract was dried over anhydrous Na_2_SO_4_ and concentrated under reduced pressure. The crude product was purified by column chromatography and the title compound (I) was obtained as dull brown crystalline solid in 61–79^th^ fraction (20 ml each) using 2.5% CHCl_3_ (3 × 500 ml) as mobile phase. Yield: 15%.

### Refinement

The H-atoms were positioned geometrically with (O–H = 0.82, C–H = 0.93 Å) and treated as riding with *U*_iso_(H) = 1.2*U*_eq_(C, O).

### Crystal data

**Table:**

C_10_H_6_N_2_O_5_	*F*(000) = 480
*M**_r_* = 234.17	*D*_x_ = 1.647 Mg m^−^^3^
Monoclinic, *P*2_1_/*n*	Mo *K*α radiation, λ = 0.71073 Å
Hall symbol: -P 2yn	Cell parameters from 1058 reflections
*a* = 7.0512 (10) Å	θ = 2.5–25.1°
*b* = 16.3541 (19) Å	µ = 0.14 mm^−^^1^
*c* = 8.7988 (10) Å	*T* = 296 K
β = 111.452 (6)°	Needle, brown
*V* = 944.4 (2) Å^3^	0.32 × 0.14 × 0.12 mm
*Z* = 4	

### Data collection

**Table:**

Bruker Kappa APEXII CCD diffractometer	1684 independent reflections
Radiation source: fine-focus sealed tube	1058 reflections with *I* > 2σ(*I*)
graphite	*R*_int_ = 0.052
Detector resolution: 8.20 pixels mm^-1^	θ_max_ = 25.1°, θ_min_ = 2.5°
ω scans	*h* = −7→8
Absorption correction: multi-scan (*SADABS*; Bruker, 2005)	*k* = −19→19
*T*_min_ = 0.978, *T*_max_ = 0.982	*l* = −10→10
6868 measured reflections	

### Refinement

**Table:**

Refinement on *F*^2^	Primary atom site location: structure-invariant direct methods
Least-squares matrix: full	Secondary atom site location: difference Fourier map
*R*[*F*^2^ > 2σ(*F*^2^)] = 0.042	Hydrogen site location: inferred from neighbouring sites
*wR*(*F*^2^) = 0.119	H-atom parameters constrained
*S* = 1.00	*w* = 1/[σ^2^(*F*_o_^2^) + (0.059*P*)^2^] where *P* = (*F*_o_^2^ + 2*F*_c_^2^)/3
1684 reflections	(Δ/σ)_max_ < 0.001
155 parameters	Δρ_max_ = 0.18 e Å^−^^3^
0 restraints	Δρ_min_ = −0.17 e Å^−^^3^

### Special details

**Table:**

Geometry. All e.s.d.'s (except the e.s.d. in the dihedral angle between two l.s. planes) are estimated using the full covariance matrix. The cell e.s.d.'s are taken into account individually in the estimation of e.s.d.'s in distances, angles and torsion angles; correlations between e.s.d.'s in cell parameters are only used when they are defined by crystal symmetry. An approximate (isotropic) treatment of cell e.s.d.'s is used for estimating e.s.d.'s involving l.s. planes.
Refinement. Refinement of *F*^2^ against ALL reflections. The weighted *R*-factor *wR* and goodness of fit *S* are based on *F*^2^, conventional *R*-factors *R* are based on *F*, with *F* set to zero for negative *F*^2^. The threshold expression of *F*^2^ > σ(*F*^2^) is used only for calculating *R*-factors(gt) *etc*. and is not relevant to the choice of reflections for refinement. *R*-factors based on *F*^2^ are statistically about twice as large as those based on *F*, and *R*- factors based on ALL data will be even larger.

### Fractional atomic coordinates and isotropic or equivalent isotropic displacement parameters (Å^2^)

**Table:**

	*x*	*y*	*z*	*U*_iso_*/*U*_eq_	
O1	0.3884 (3)	0.45828 (13)	0.10925 (19)	0.0656 (6)	
O2	0.6120 (3)	0.36519 (11)	0.1294 (2)	0.0663 (6)	
O3	0.8511 (3)	0.26242 (11)	0.6881 (2)	0.0762 (7)	
O4	1.0038 (3)	0.34485 (11)	0.8845 (2)	0.0735 (6)	
O5	0.9984 (3)	0.49989 (10)	0.83341 (16)	0.0500 (5)	
H5A	1.0356	0.4587	0.8896	0.060*	
N1	0.5444 (3)	0.42063 (12)	0.1860 (2)	0.0441 (5)	
N2	0.8995 (3)	0.33110 (13)	0.7401 (3)	0.0497 (6)	
C1	0.8052 (3)	0.54453 (12)	0.5693 (2)	0.0313 (5)	
C2	0.8467 (4)	0.62585 (14)	0.6216 (3)	0.0415 (6)	
H2	0.9197	0.6370	0.7312	0.050*	
C3	0.7809 (4)	0.68880 (14)	0.5131 (3)	0.0465 (7)	
H3	0.8077	0.7426	0.5489	0.056*	
C4	0.6744 (4)	0.67246 (15)	0.3500 (3)	0.0487 (7)	
H4	0.6329	0.7157	0.2766	0.058*	
C5	0.6283 (4)	0.59409 (15)	0.2936 (3)	0.0419 (6)	
H5	0.5557	0.5848	0.1832	0.050*	
C6	0.6905 (3)	0.52753 (13)	0.4024 (2)	0.0313 (5)	
C7	0.6553 (3)	0.44389 (13)	0.3569 (2)	0.0334 (5)	
C8	0.7224 (3)	0.38182 (13)	0.4646 (2)	0.0373 (6)	
H8	0.6948	0.3279	0.4299	0.045*	
C9	0.8335 (3)	0.39941 (13)	0.6284 (2)	0.0354 (6)	
C10	0.8816 (3)	0.47868 (13)	0.6824 (2)	0.0345 (5)	

### Atomic displacement parameters (Å^2^)

**Table:**

	*U*^11^	*U*^22^	*U*^33^	*U*^12^	*U*^13^	*U*^23^
O1	0.0468 (12)	0.0892 (14)	0.0412 (10)	0.0043 (11)	−0.0071 (9)	−0.0067 (9)
O2	0.0875 (16)	0.0570 (12)	0.0489 (11)	0.0026 (11)	0.0184 (10)	−0.0192 (9)
O3	0.110 (2)	0.0391 (11)	0.0731 (13)	−0.0007 (11)	0.0256 (12)	0.0117 (9)
O4	0.0881 (16)	0.0695 (13)	0.0441 (11)	−0.0059 (11)	0.0017 (11)	0.0225 (9)
O5	0.0560 (12)	0.0565 (10)	0.0260 (9)	−0.0001 (9)	0.0012 (8)	0.0046 (7)
N1	0.0459 (14)	0.0488 (12)	0.0320 (11)	−0.0085 (11)	0.0075 (10)	−0.0052 (9)
N2	0.0521 (15)	0.0483 (14)	0.0490 (13)	0.0019 (11)	0.0190 (11)	0.0158 (10)
C1	0.0258 (13)	0.0386 (12)	0.0291 (11)	0.0020 (10)	0.0096 (9)	−0.0002 (9)
C2	0.0405 (15)	0.0446 (14)	0.0385 (13)	−0.0023 (11)	0.0132 (11)	−0.0055 (11)
C3	0.0493 (17)	0.0376 (14)	0.0519 (15)	0.0043 (12)	0.0175 (13)	0.0013 (11)
C4	0.0490 (17)	0.0448 (15)	0.0489 (15)	0.0094 (12)	0.0139 (13)	0.0142 (12)
C5	0.0368 (15)	0.0526 (15)	0.0317 (12)	0.0054 (12)	0.0070 (10)	0.0065 (11)
C6	0.0235 (12)	0.0401 (12)	0.0298 (11)	0.0026 (10)	0.0093 (9)	0.0016 (9)
C7	0.0267 (13)	0.0435 (13)	0.0272 (11)	−0.0026 (10)	0.0066 (9)	−0.0030 (10)
C8	0.0347 (14)	0.0384 (13)	0.0388 (13)	−0.0021 (11)	0.0137 (11)	−0.0017 (10)
C9	0.0337 (14)	0.0404 (13)	0.0321 (12)	0.0021 (10)	0.0120 (10)	0.0083 (10)
C10	0.0307 (13)	0.0474 (14)	0.0247 (11)	0.0008 (11)	0.0093 (10)	0.0021 (10)

### Geometric parameters (Å, °)

**Table:**

O1—N1	1.225 (2)	C2—H2	0.9300
O2—N1	1.213 (2)	C3—C4	1.381 (3)
O3—N2	1.214 (3)	C3—H3	0.9300
O4—N2	1.234 (3)	C4—C5	1.370 (3)
O5—C10	1.328 (2)	C4—H4	0.9300
O5—H5A	0.8200	C5—C6	1.409 (3)
N1—C7	1.469 (3)	C5—H5	0.9300
N2—C9	1.448 (3)	C6—C7	1.421 (3)
C1—C2	1.403 (3)	C7—C8	1.351 (3)
C1—C6	1.421 (3)	C8—C9	1.395 (3)
C1—C10	1.431 (3)	C8—H8	0.9300
C2—C3	1.365 (3)	C9—C10	1.380 (3)
			
C10—O5—H5A	109.5	C4—C5—C6	120.2 (2)
O2—N1—O1	123.9 (2)	C4—C5—H5	119.9
O2—N1—C7	118.2 (2)	C6—C5—H5	119.9
O1—N1—C7	117.9 (2)	C5—C6—C7	124.98 (19)
O3—N2—O4	122.4 (2)	C5—C6—C1	118.0 (2)
O3—N2—C9	118.8 (2)	C7—C6—C1	117.00 (19)
O4—N2—C9	118.8 (2)	C8—C7—C6	123.06 (19)
C2—C1—C6	119.75 (19)	C8—C7—N1	116.25 (19)
C2—C1—C10	120.33 (19)	C6—C7—N1	120.68 (18)
C6—C1—C10	119.90 (19)	C7—C8—C9	119.3 (2)
C3—C2—C1	120.6 (2)	C7—C8—H8	120.3
C3—C2—H2	119.7	C9—C8—H8	120.3
C1—C2—H2	119.7	C10—C9—C8	121.60 (19)
C2—C3—C4	119.8 (2)	C10—C9—N2	120.89 (19)
C2—C3—H3	120.1	C8—C9—N2	117.5 (2)
C4—C3—H3	120.1	O5—C10—C9	125.07 (19)
C5—C4—C3	121.6 (2)	O5—C10—C1	115.92 (19)
C5—C4—H4	119.2	C9—C10—C1	119.00 (18)
C3—C4—H4	119.2		
			
C6—C1—C2—C3	−1.1 (3)	O1—N1—C7—C6	−44.9 (3)
C10—C1—C2—C3	177.5 (2)	C6—C7—C8—C9	−0.7 (3)
C1—C2—C3—C4	−0.7 (4)	N1—C7—C8—C9	178.4 (2)
C2—C3—C4—C5	1.5 (4)	C7—C8—C9—C10	−1.9 (3)
C3—C4—C5—C6	−0.4 (4)	C7—C8—C9—N2	179.0 (2)
C4—C5—C6—C7	−178.5 (2)	O3—N2—C9—C10	178.8 (2)
C4—C5—C6—C1	−1.4 (3)	O4—N2—C9—C10	−1.5 (3)
C2—C1—C6—C5	2.1 (3)	O3—N2—C9—C8	−2.1 (3)
C10—C1—C6—C5	−176.42 (19)	O4—N2—C9—C8	177.6 (2)
C2—C1—C6—C7	179.44 (19)	C8—C9—C10—O5	−175.0 (2)
C10—C1—C6—C7	0.9 (3)	N2—C9—C10—O5	4.1 (3)
C5—C6—C7—C8	178.2 (2)	C8—C9—C10—C1	3.8 (3)
C1—C6—C7—C8	1.1 (3)	N2—C9—C10—C1	−177.11 (19)
C5—C6—C7—N1	−0.8 (3)	C2—C1—C10—O5	−2.9 (3)
C1—C6—C7—N1	−177.9 (2)	C6—C1—C10—O5	175.7 (2)
O2—N1—C7—C8	−43.7 (3)	C2—C1—C10—C9	178.2 (2)
O1—N1—C7—C8	136.1 (2)	C6—C1—C10—C9	−3.2 (3)
O2—N1—C7—C6	135.4 (2)		

### Hydrogen-bond geometry (Å, °)

**Table:**

*D*—H···*A*	*D*—H	H···*A*	*D*···*A*	*D*—H···*A*
O5—H5A···O1^i^	0.82	2.53	3.006 (3)	118
O5—H5A···O4	0.82	1.87	2.573 (2)	142
O5—H5A···N2	0.82	2.47	2.892 (3)	113
C5—H5···O1	0.93	2.35	2.902 (3)	118

Symmetry codes: (i) *x*+1, *y*, *z*+1.

### Table 2 π–π interactions (Å, °)

Cg1 and Cg2 are the centroids of the  C1–C6 and C1/C6–C10 rings, respectively. ipd is the mean interplanar distance (distance from one plane to the neighbouring centroid) and sa is the slippage angle (angle subtended by the intercentroid vector to the plane normal). For details, see Janiak (2000)

**Table:**

	Cg···Cg	ipd	sa
Cg1···Cg2^i^	3.6296 (15)	3.365	1.305
Cg1···Cg2^ii^	3.8104 (15)	3.552	1.323
Cg2···Cg2^i^	3.6513 (14)	3.378	1.386

Symmetry codes: (i) 1-x, 1-y, 1-z, (ii) 2-x, 1-y, 1-z.
